# Effect of Silver Deposition on the Material Characteristics and Photocatalytic Activity of Barium Titanates

**DOI:** 10.3390/nano16201322

**Published:** 2026-10-20

**Authors:** Áron Ágoston, Eszter Kanász, Szabolcs Kocsis Szürke, Mahsa Abedi, Karolina Solymos, Csaba Bús, Milica Todea, Ágota Deák, Tamás Gyulavári, Ákos Kukovecz, Zoltán Kónya, Zsolt Pap

**Affiliations:** 1 Department of Physical Chemistry and Materials Sciences, University of Szeged, Aradi v. Sqr. 1, 6720 Szeged, Hungary; agostona@chem.u-szeged.hu (Á.Á.); agotadeak@chem.u-szeged.hu (Á.D.); 2 Department of Applied and Environmental Chemistry, University of Szeged, Rerrich Béla Sqr. 1, 6720 Szeged, Hungary; kanasz.eszter@gmail.com (E.K.); mahsa@chem.u-szeged.hu (M.A.); kakos@chem.u-szeged.hu (Á.K.); konya@chem.u-szeged.hu (Z.K.); pzsolt@chem.u-szeged.hu (Z.P.); 3 Vehicle Industry Research Center, Széchenyi István University, Egyetem tér 1. 303, 9026 Győr, Hungary; kocsis.szurke.szabolcs@sze.hu; 4 Department of Physical and Environmental Geography, University of Szeged, Egyetem Str. 2-6, 6722 Szeged, Hungary; solymoskarolina@geo.u-szeged.hu; 5 Department of Molecular and Analytical Chemistry, University of Szeged, Dóm Sqr. 7-8, 6720 Szeged, Hungary; bcsaba0312@gmail.com; 6 Faculty of Nursing and Health Sciences, Iuliu Hațieganu University of Medicine and Pharmacy, Victor Babeș 8, RO-400012 Cluj-Napoca, Romania; militodea@yahoo.com; 7 Nanostructured Materials and Bio-Nano-Interfaces Center, Interdisciplinary Research Institute on Bio-Nano-Sciences, Babes-Bolyai University, Treboniu Laurian 42, RO-400271 Cluj-Napoca, Romania; 8 Institute of Research-Development-Innovation in Applied Natural Sciences, Babes-Bolyai University, Fântânele Str. 30, RO-400294 Cluj-Napoca, Romania

**Keywords:** photocatalysis, barium titanate, silver, noble metal deposition, phenol, metal size effect

## Abstract

Silver (0–5 wt%) was reduced on the surface of barium titanates (BaTiO_3_) to investigate its effect on the properties and photocatalytic activity of the support. Samples were characterized by X-ray diffractometry, diffuse reflectance spectroscopy, photoluminescence, transmission electron microscopy, photocurrent density, and linear sweep voltammetry. Their photocatalytic activity was evaluated by phenol degradation under UV light irradiation. A commercial BaTiO_3_ without silver was used as a reference. Nominal silver amounts were consistent with the measured ones in the samples containing 0–3 wt% silver; however, at high nominal amounts (4–5 wt%), the measured silver content decreased significantly (~1.3 wt%). Samples containing larger silver particles had higher photoactivity than samples containing smaller silver particles. The sample containing 5 wt% silver performed the best, reaching a phenol degradation rate of 2.10 × 10^−4^ s^−1^, over an order of magnitude higher than that of the non-silver-containing reference sample (1.47 × 10^−5^ s^−1^). The enhanced photoactivity was associated with the larger silver particles covering less of the BaTiO_3_ surface, leaving more active sites available, and with the silver hindering charge recombination by trapping the electrons and thus increasing the efficiency of holes.

## 1. Introduction

Barium titanate (BaTiO_3_) has attracted significant attention due to its beneficial properties, such as potential dielectricity [1], potential ferroelectricity [2,3], and piezoelectricity [3,4,5]. Application possibilities such as energy storage [6], CO_2_ sensing [7], glucose sensing [8], gate insulation [9], and capacitor development [10] have already been reported in the literature. Using BaTiO_3_ as a photocatalyst for environmental remediation is another promising application [3,4,5,11]. Although the band gaps reported in the literature vary between 3.0 [12] and 3.6 eV [4], it can be ascertained that BaTiO_3_ can only be excited efficiently by UV photons, and its photocatalytic activity often does not reach the threshold required for applications. There are many possibilities to enhance photoactivity, including doping with various elements, (noble) metal deposition, morphological modifications, construction of composites and heterostructures/heterojunctions, and dye sensitization [13,14,15].

Compared to many other photocatalysts (e.g., ZnO and Fe_2_O_3_), it is preferable to modify BaTiO_3_ with silver because its conduction band minimum is more negative than that of most commercial oxide photocatalysts (approximately −0.8 V for BaTiO_3_ [16], −0.5 V for ZnO [17], −0.65 V for TiO_2_ [18], and 0.3 V for Fe_2_O_3_ [19]). A more negative conduction band potential means that excited electrons have a higher reduction potential and are therefore more likely to be trapped by silver.

Enhancing the photoactivity of BaTiO_3_ via Ag deposition is a promising approach [3,4,5,11,20]. The surface plasmon resonance effect of Ag nanoparticles can enable the absorbance of visible light [3,5,20], promote charge separation and electron trapping [21], and increase the lifetime of photogenerated charge carriers [5]. The mechanisms resulting in enhanced photoactivity include (i) efficient electron transfer between the catalyst and Ag nanoparticles, (ii) electron transfer from Ag to the catalyst mediated by Ag surface plasmons, (iii) nonirradiation decay of Ag surface plasmons causing localized heating of Ag, and (iv) Ag nanoparticles emitting visible photons to the catalyst through the radiative decay of surface plasmon states [22,23]. Xu et al. reported that the size of Ag nanoparticles is also an important parameter that can influence the photocatalytic activity of BaTiO_3_ [5].

In this paper, we study the effect of Ag deposition (0–5 wt%) and the size of Ag (nano)particles on the properties of BaTiO_3_ photocatalysts. Changes in the structural, optical, and electrochemical properties of the catalysts are investigated. In the literature, dyes are used most frequently to evaluate photoactivity such as methylene blue [24], methyl orange [4,11], and rhodamine B [3,5]. We use phenol to determine the photoactivity of Ag-modified BaTiO_3_ photocatalysts as, to the best of our knowledge, it has not been investigated so far. Emphasis is also put on the quantification of exciton lifetime modified by the presence of silver.

## 2. Results and Discussion

### 2.1. Characterization of the Photocatalysts

#### 2.1.1. Crystal Phase Composition (XRD) and Silver Content (XRF)

**Figure 1:**
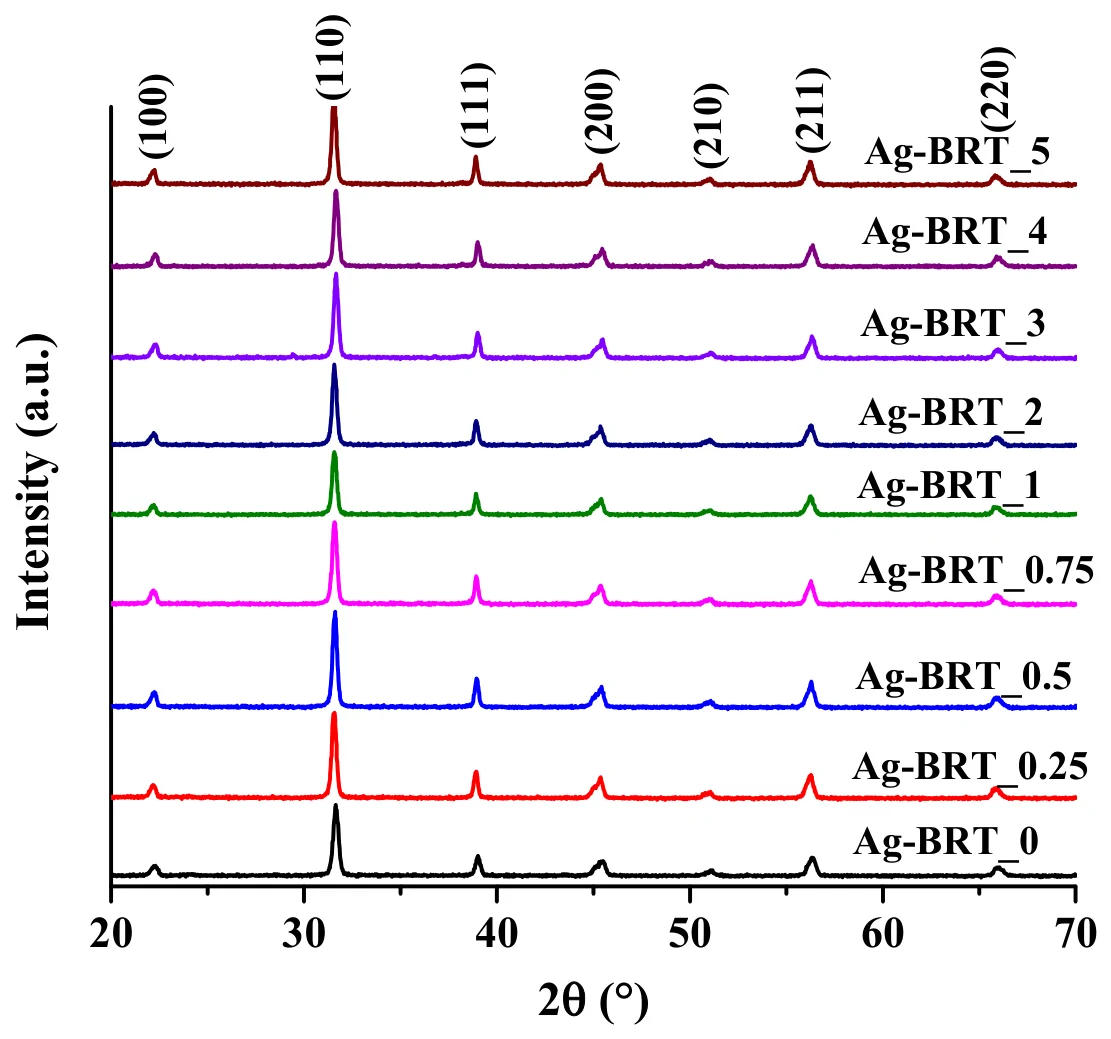
XRD patterns of the investigated samples.

The XRD patterns obtained (Figure 1) show the (100), (110), (111), (200), (210), (211), and (220) crystallographic planes of BaTiO_3_ at 22.3°, 31.7°, 38.9°, 45.2°, 50.9°, 56.1°, and 65.8°, respectively. The diffraction peak at 45.2° splits into (200) and (002) reflections, which is characteristic of tetragonal BaTiO_3_ [25] with ferroelectric properties [26]. The XRD patterns show that Ag deposition does not cause observable changes in the crystalline composition of the samples. Diffraction peaks of Ag or Ag-containing compounds are absent either because they are not crystalline or their amount does not reach the limit of detection of the diffractometer. The crystallization of silver is an energy-demanding process. This energy is mostly provided by heat treatment. Amorphous silver nanoparticles turn into crystalline ones at a temperature of approximately 340 °C [27]. Heat treatment was not applied during synthesis, so the silver on the surface of BaTiO_3_ is amorphous. The primary crystallite sizes of the BaTiO_3_ support, calculated via the Scherrer equation, were determined to be ~28 nm across all samples. As no Bragg reflections for Ag were detectable in the XRD patterns, the silver nanoparticle sizes were evaluated directly based on TEM micrographs.

The amount of Ag successfully deposited on the samples was evaluated by X-ray fluorescence (XRF) measurements. Table 1 shows that the Ag amount of the samples increases with the nominal Ag amount used during their synthesis and that they are in reasonably good agreement with each other until they reach 3 wt% Ag. However, at higher nominal loadings of 4 and 5 wt%, the measured Ag content unexpectedly decreases to ~1.45 wt% and ~1.22 wt%, respectively. This discrepancy is most likely directly related to the reduction kinetics and nanoparticle growth behavior during synthesis. As the absolute amount of NaBH_4_ scales with higher nominal Ag loadings, the rapid reduction rate promotes the growth of larger Ag nanoparticles. Due to steric hindrance and lower interfacial binding stability, these larger Ag particles cannot fully anchor to the BaTiO_3_ surface and are subsequently removed during the centrifugation/washing process. This result is discussed further in the next section.

#### 2.1.2. Optical Properties (DRS and PL)

**Figure 2:**
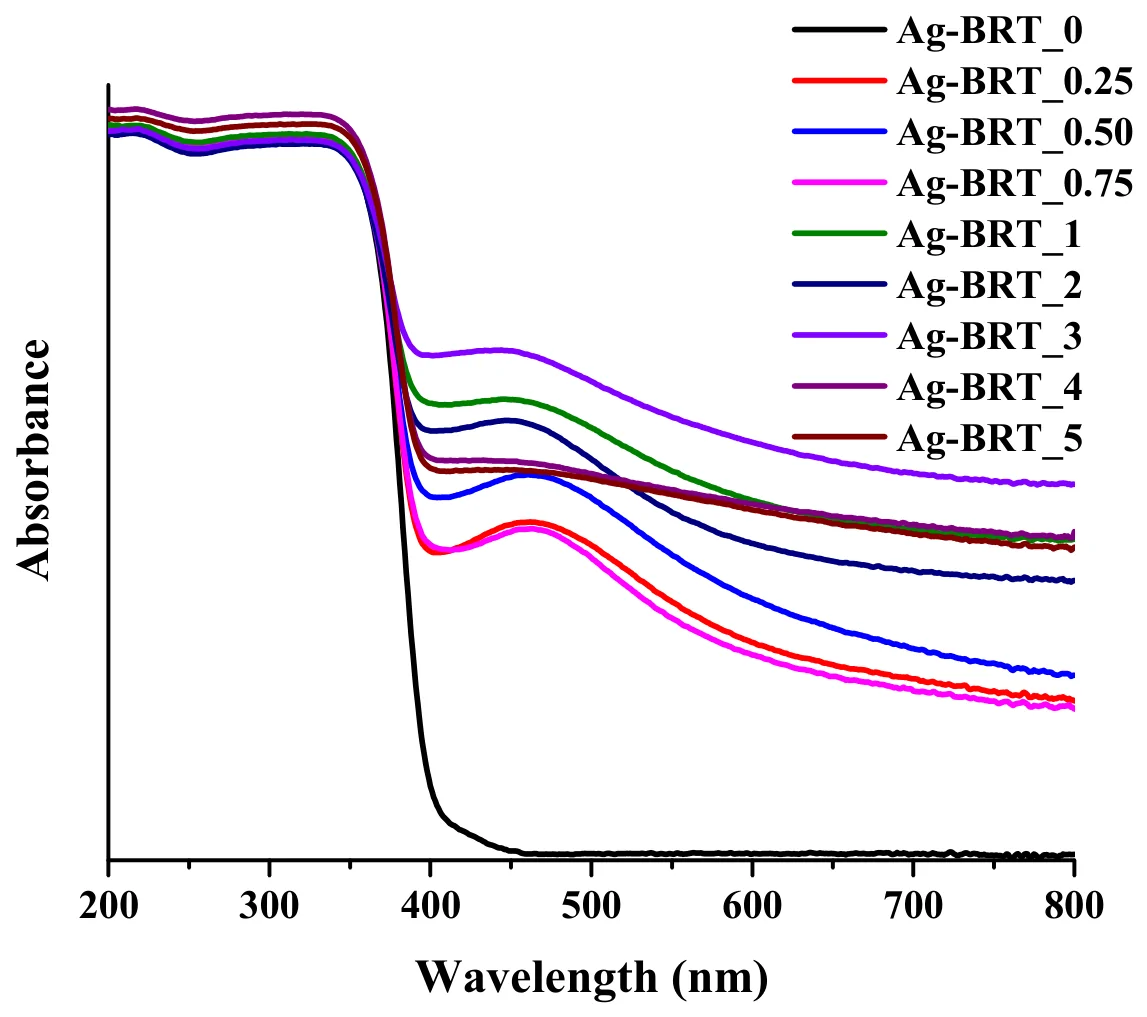
DR spectra of the investigated samples.

The presence of metallic silver nanoparticles is further supported by diffuse reflectance spectroscopy (DRS) measurements (Figure 2). Deposited Ag nanoparticles exhibit characteristic broad absorption across the visible spectrum (400–800 nm) due to localized surface plasmon resonance (LSPR). Some samples exhibit a distinct local absorption maximum in the 400–550 nm region, which is characteristic of the LSPR of metallic silver (Ag^0^) nanoparticles. The appearance of plasmon resonance bands is associated with Ag on the surface; however, they disappear in Ag-BRT_4 and Ag-BRT_5. If the BaTiO_3_ support did not affect the silver particles, they would have plasmonic properties by themselves at nominal silver concentrations exceeding 4 wt%. This may be attributed to the dielectric screening effect originating from the high refractive index of BaTiO_3_ [28]. In accordance with Mie theory, as the contact area between the metal and the support increases with increasing particle size, the LSPR absorption band undergoes a significant red-shift and significantly broadens spectrally. This broadened absorption profile results in a reflection and extinction across the entire visible spectrum, which makes the plasmonic peak in the DRS spectrum undetectable and imparts a gray appearance to the sample. BRT_4 and Ag-BRT_5 are different from the rest of the sample series in more than just the nominal Ag amount, as their size is also different, making them less comparable to the other samples.

As shown in Figure 2, while the fundamental absorption edge of BaTiO_3_ undergoes minimal shift, the reflectance spectra transformed via the Kubelka–Munk method and the calculated apparent band gaps from the Tauc plots (Figure S1) exhibit a downward trend from 3.19 eV to 3.10 eV from sample Ag-BRT_0 to Ag-BRT_3, while the value for samples Ag-BRT_4–5 is 3.16 eV, which is consistent with the literature [29]. It should be noted that this variation does not reflect a true change in the intrinsic bulk band gap of BaTiO_3_. Instead, the observed apparent decrease can be attributed to the size and morphology of the deposited silver nanoparticles. The small-sized silver nanoparticles provide a larger interfacial contact area with the BaTiO_3_ surface, and, due to the difference in Fermi levels, a Schottky barrier is formed. This induces local band bending and the creation of interfacial defect states and localized energy levels, which influence the optical absorption profile and the Tauc plot fitting, resulting in an apparent decrease in the calculated band gap values.

**Figure 3:**
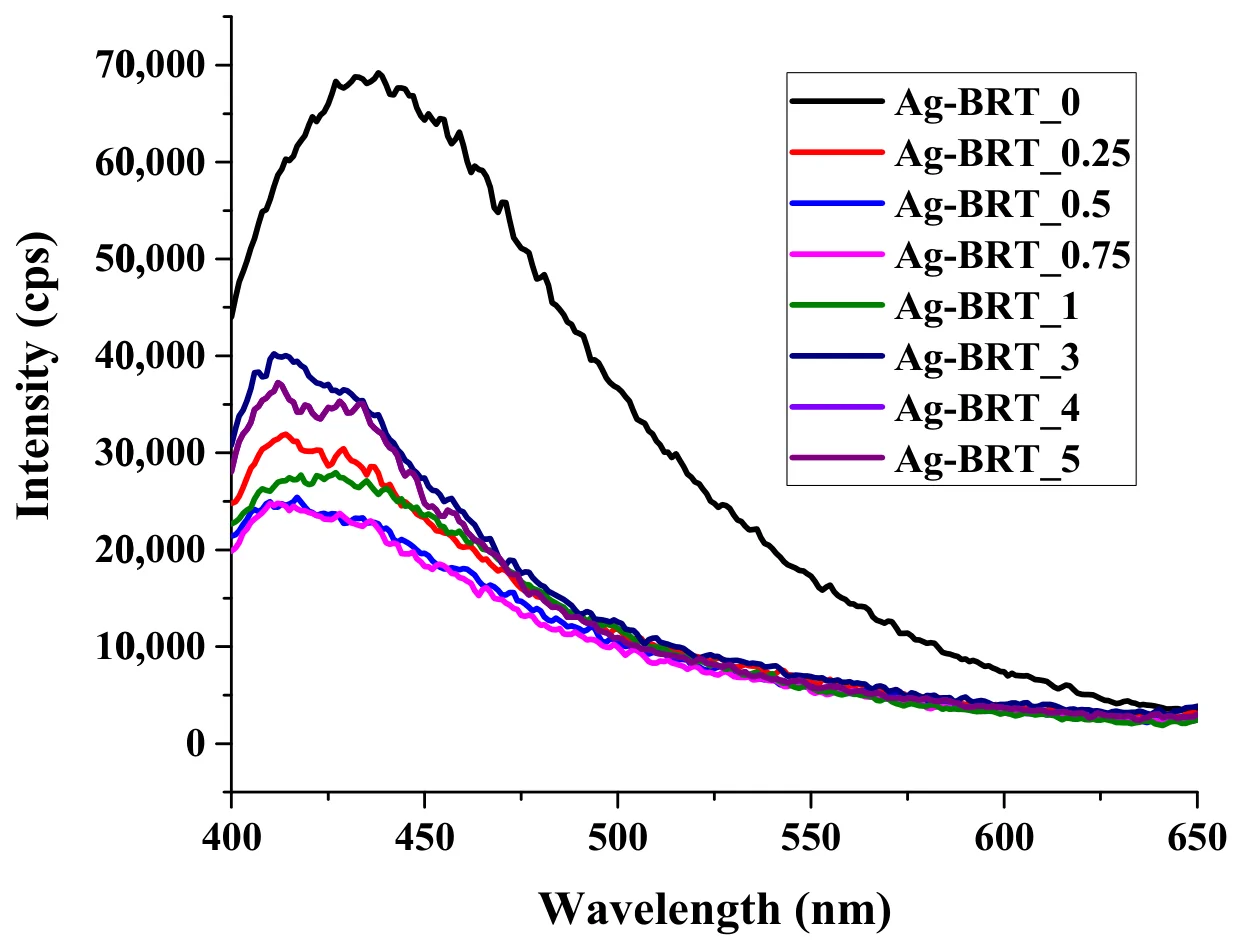
PL spectra of the investigated samples.

**Figure 4:**
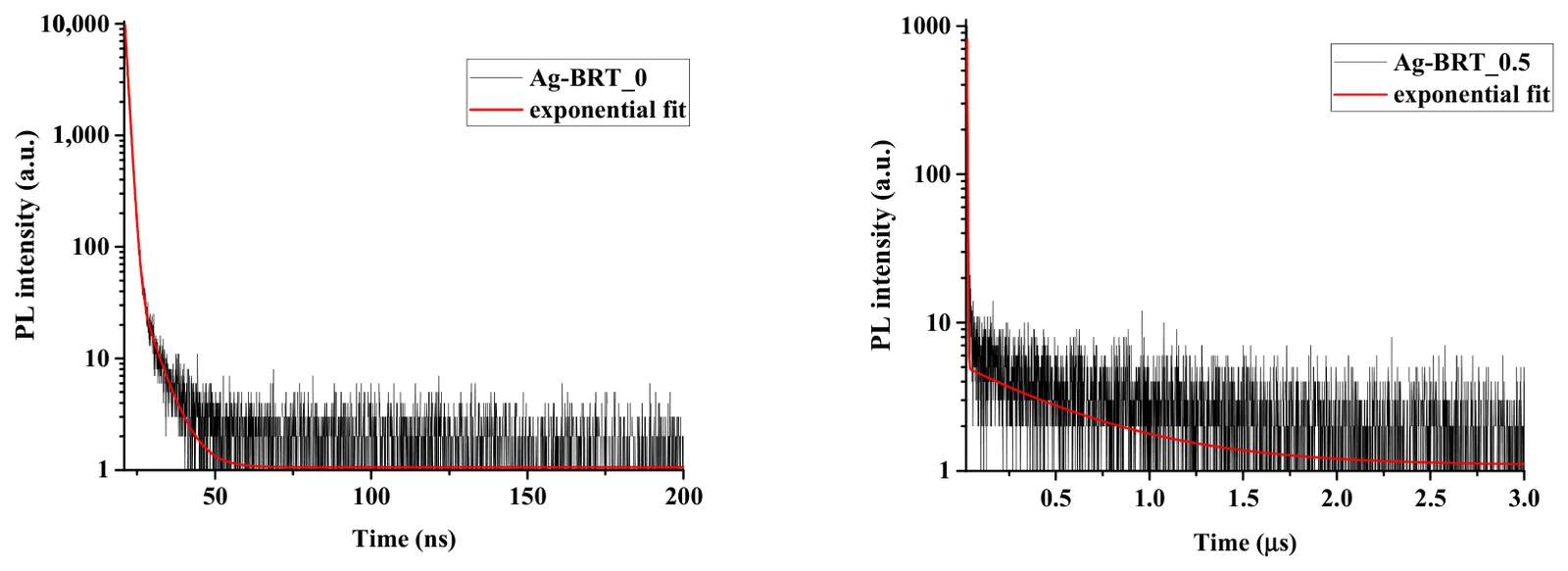

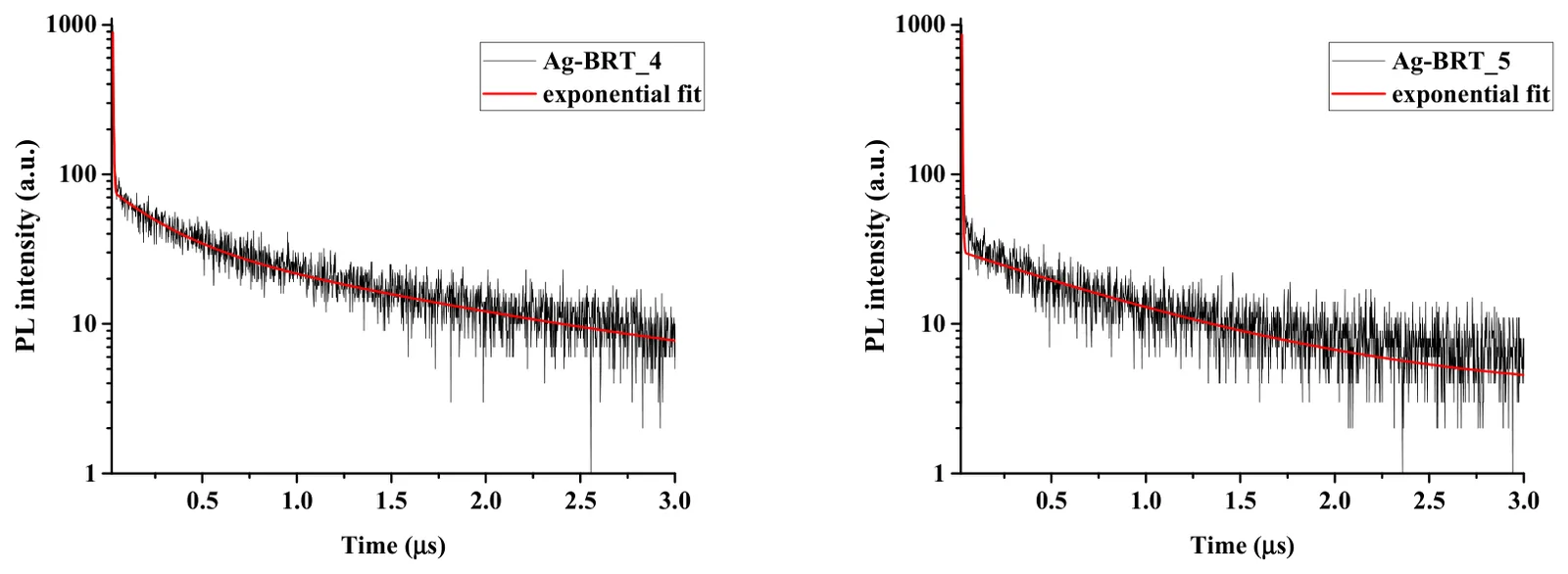
Time-resolved PL spectra of the investigated samples.

Charge recombination was investigated by photoluminescence (PL) measurements, and the results are shown in Figure 3. Recombination associated with photon emission was successfully restricted as a result of Ag deposition compared to the reference (Ag-BRT_0). Hence, under the conditions of the PL measurement, another type of recombination occurred, such as thermal recombination. It is known that excitons must possess a sufficient lifespan for photocatalytic oxidation and reduction. Since the Ag in the Ag-containing samples can trap electrons, which thus do not recombine with photogenerated holes via photon emission, enhanced photocatalytic activity was expected in these samples [30]. The time-resolved PL results showed that the lifetime of excitons in the samples containing larger silver particles was significantly longer than that in the reference barium titanate. The average lifetime of PL in the case of Ag-BRT_0 was 0.92 ns. The Ag-BRT_0.5 sample was also measured to investigate whether silver in such a small size and low amount had an effect. In this sample, the average lifetime of PL was 3.60 ns, so despite the low amount of silver, its effect on lifetime is significant. The average lifetimes of PL for Ag-BRT_4 and Ag-BRT_5 were 18.21 ns and 42.8 ns, respectively. The spectra of the samples are shown in Figure 4, while the fitting model and related parameters (including goodness-of-fit parameters; Figure S2) are summarized in the Supplementary Materials.

#### 2.1.3. Transmission Electron Microscopy (TEM) Measurements and X-Ray Photoelectron Spectroscopy (XPS) Analysis

**Figure 5:**
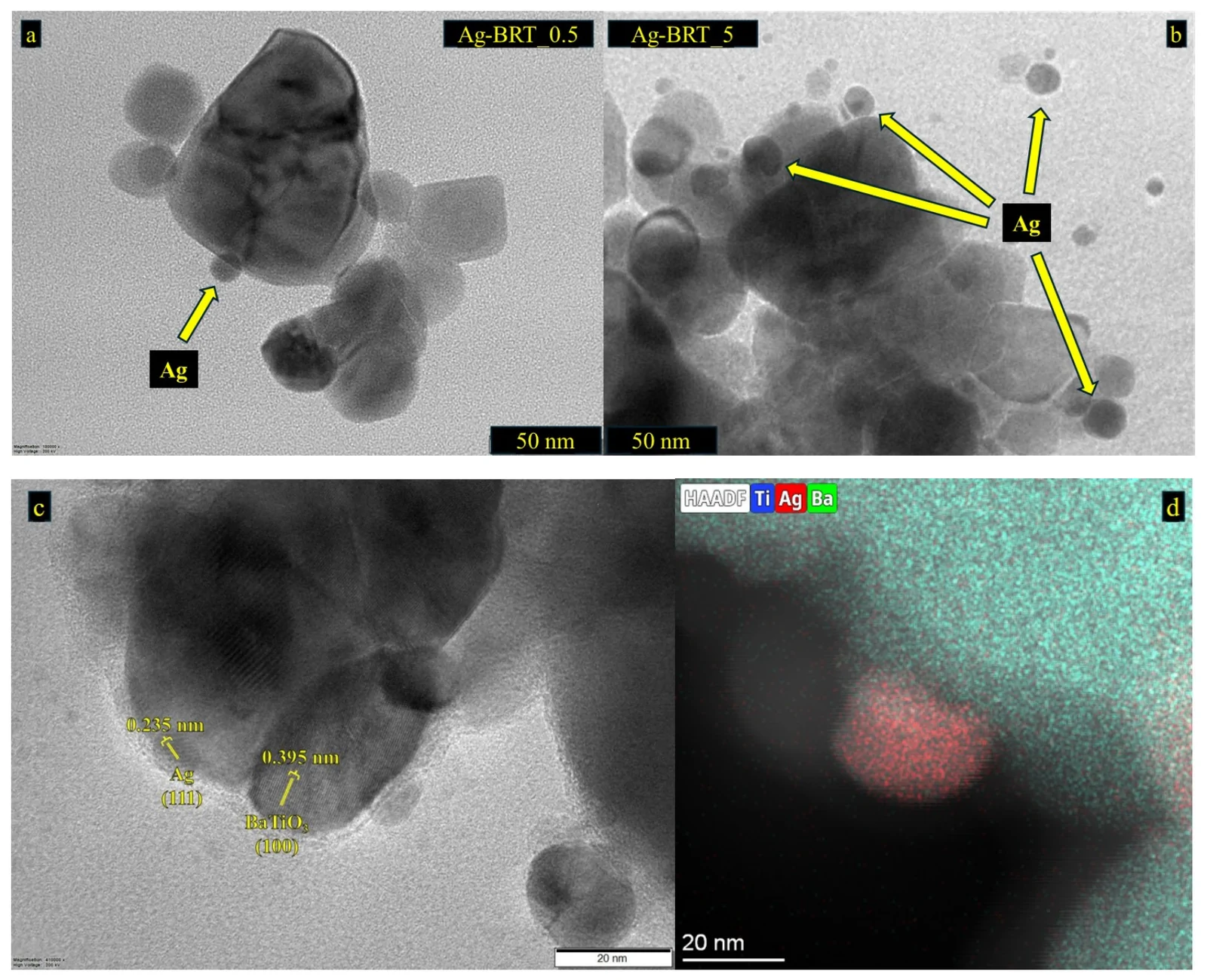

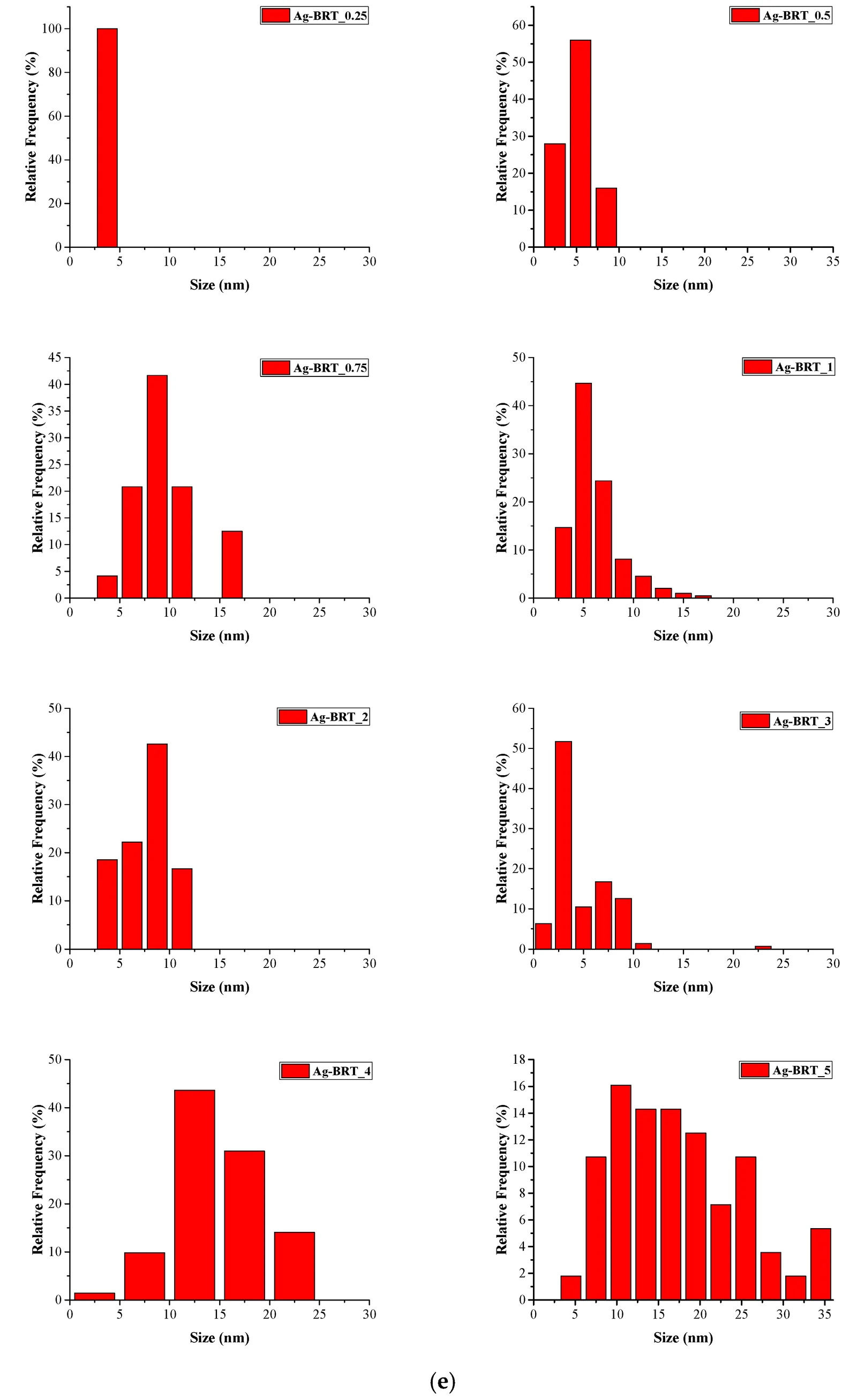
TEM images of Ag-BRT_0.5 (**a**) and Ag-BRT_5 (**b**). The d spacing of Ag and BaTiO_3_ (**c**) and a representative STEM-EDS map showing the presence of silver on the BaTiO_3_ support (**d**). Size distribution histograms of silver in all Ag-BRT samples (**e**).

To verify the size assumptions described in Section 2.1.2 (i.e., samples with a higher nominal silver content have larger silver particles on the BaTiO_3_ surface), TEM images were captured. Figure 5 shows the micrographs of Ag-BRT_0.5 (Figure 5a) and Ag-BRT_5 (Figure 5b), while the TEM results of the complete sample series is shown in the Supplementary Materials (Figure S3). They show that the BaTiO_3_ support consists of partly angular, partly rounded shapes, while the significantly smaller silver nanoparticles are spherical [31]. The BaTiO_3_ support and silver particles can be easily distinguished based on their morphologies and lattice parameters (Figure 5c). Further proof of the successful modification of BaTiO_3_ with silver is demonstrated in the scanning transmission electron microscopy energy-dispersive X-ray spectroscopy (STEM-EDS) elemental map (Figure 5d). The TEM images confirm the assumption that the Ag particles on the surface of BaTiO_3_ are smaller on average for Ag-BRT_0.25–3 (i.e., Ag-BRT_0.25, Ag-BRT_0.5, Ag-BRT_0.75, Ag-BRT_1, Ag-BRT_2, and Ag-BRT_3) than for Ag-BRT_4 and Ag-BRT_5. The size of silver was calculated based on size distribution histograms. Accordingly, the average silver sizes were 3.9, 5.1, 9.5, 6.1, 7.6, 4.7, 14.9, and 18.0 nm in Ag_BRT_0.25, Ag_BRT_0.5, Ag_BRT_0.75, Ag_BRT_1, Ag_BRT_2, Ag_BRT_3, Ag_BRT_4, and Ag_BRT_5, respectively.

**Figure 6:**
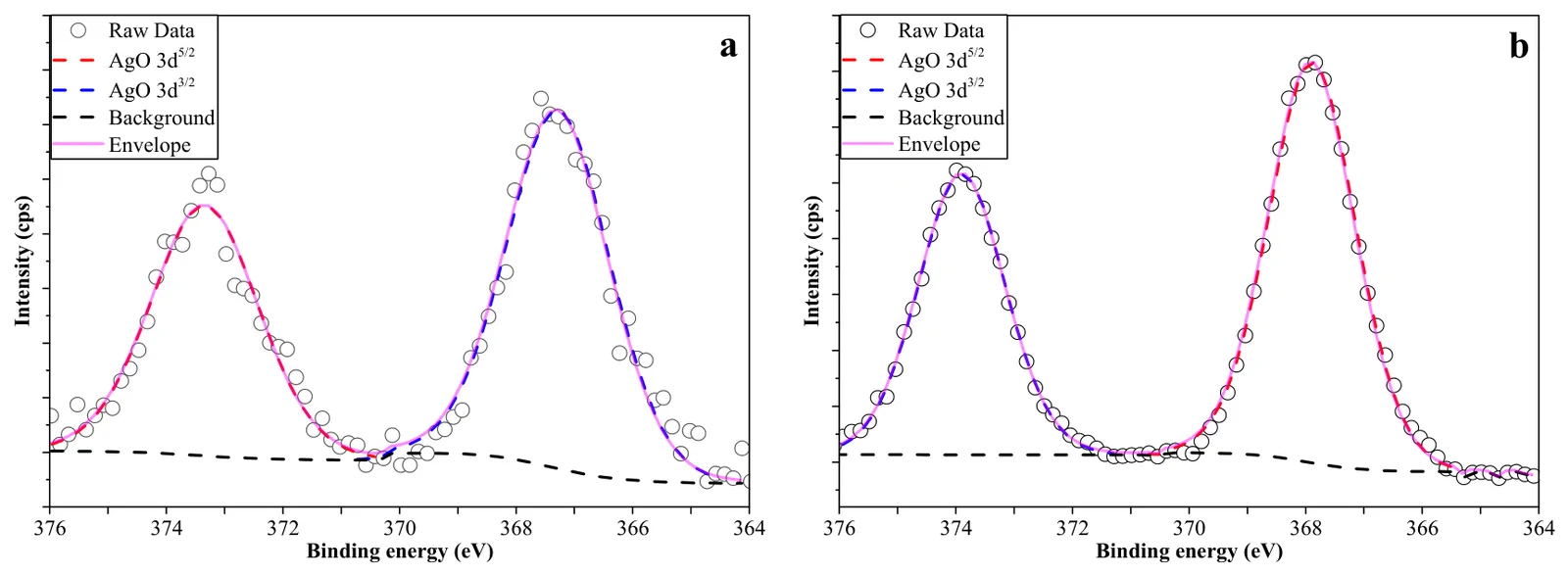
XPS spectra of Ag-BRT_1 (**a**) and Ag-BRT_5 (**b**) showing the possible presence of Ag and/or Ag^+^.

To further characterize the silver species, XPS measurements were carried out, and the Ag 3d spectra are shown in Figure 6. Owing to the low silver loading, the Ag Auger signal [32] could not be reliably detected and therefore could not be used for determining the oxidation states. Consequently, the analysis was based on the Ag 3d core-level spectra. Ag 3d_5/2_ and Ag 3d_3/2_ peaks were observed at approximately 368.2 and 374.2 eV, respectively. These binding energies may be attributed either to Ag_2_O [33] or metallic Ag [34]. The assignment of silver oxidation states based on Ag 3d binding energies remains challenging, as significant variations (up to 1.0–1.5 eV) have been reported in the literature, even for the same silver compound. In addition, silver nanoparticles are known to readily form a surface oxide layer under ambient conditions, a process that is thermodynamically favored [35]. Therefore, the presence of oxidized silver species on the nanoparticle surface is plausible. Based on the XPS results, together with the TEM observations, a realistic interpretation is that a Ag_2_O layer is present on the surface of the nanoparticles, while the nanoparticle core remains predominantly metallic Ag.

#### 2.1.4. Specific Surface Area Measurements (BET)

The specific surface area values show a slight decrease with an increasing nominal silver content (Table 1). This can be attributed to the spherical, well-fitting deposited nanoparticles starting to fill the surface irregularities of BaTiO_3_, covering the uneven surface originating from the anisotropy of BaTiO_3_. For samples Ag-BRT_4 and Ag-BRT_5, the decrease in specific surface area is more pronounced, which can be explained by the larger deposited nanoparticles (with size increase proven by other methods) clogging the pores still accessible to N_2_, in addition to covering a portion of BaTiO_3_, and the larger size of the deposited particles themselves. Due to the steric hindrance of the nanoparticles, the contact area between BaTiO_3_ and the deposited nanoparticles is smaller, which positively affects the specific surface area due to the exposed, more uneven BaTiO_3_ surface; however, the extensive pore clogging counterbalances this effect. The BET plots are shown in Figure S4.

#### 2.1.5. Electrochemical Properties

**Figure 7:**
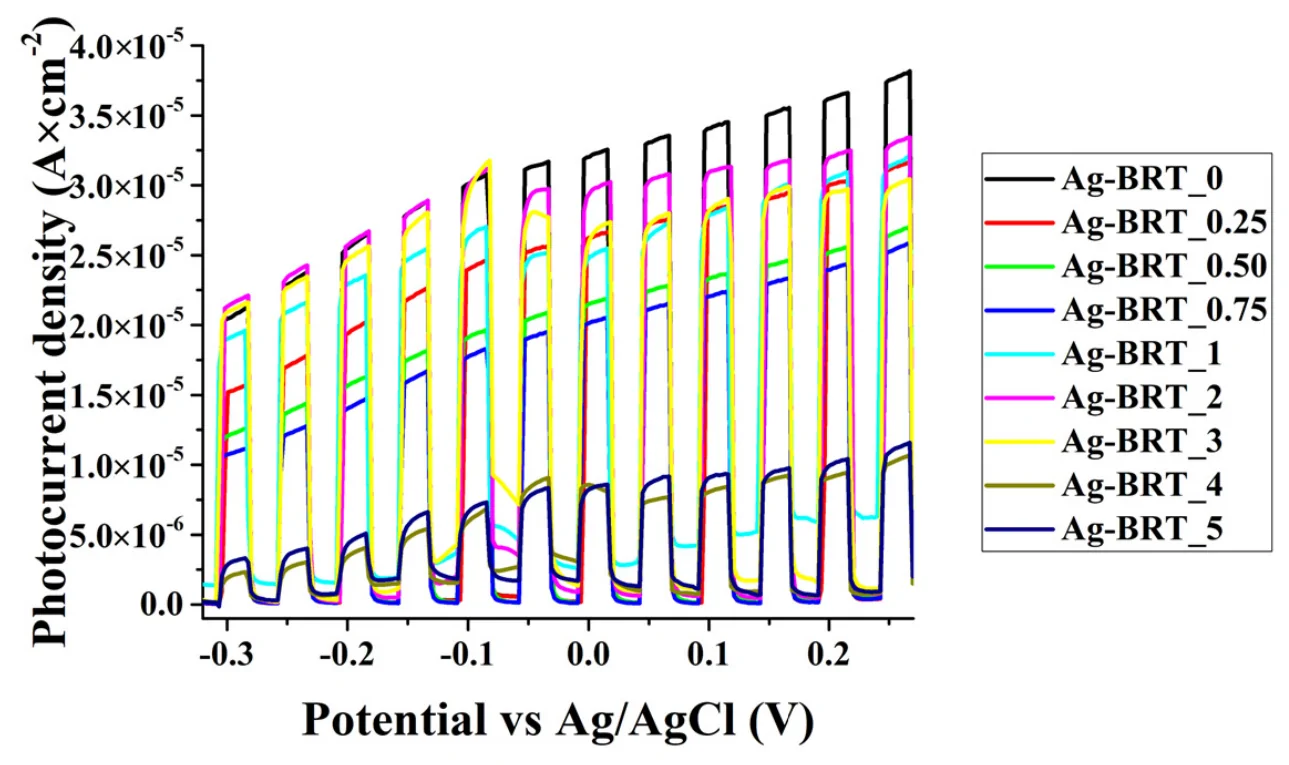
Voltammogram of the investigated samples.

**Figure 8:**
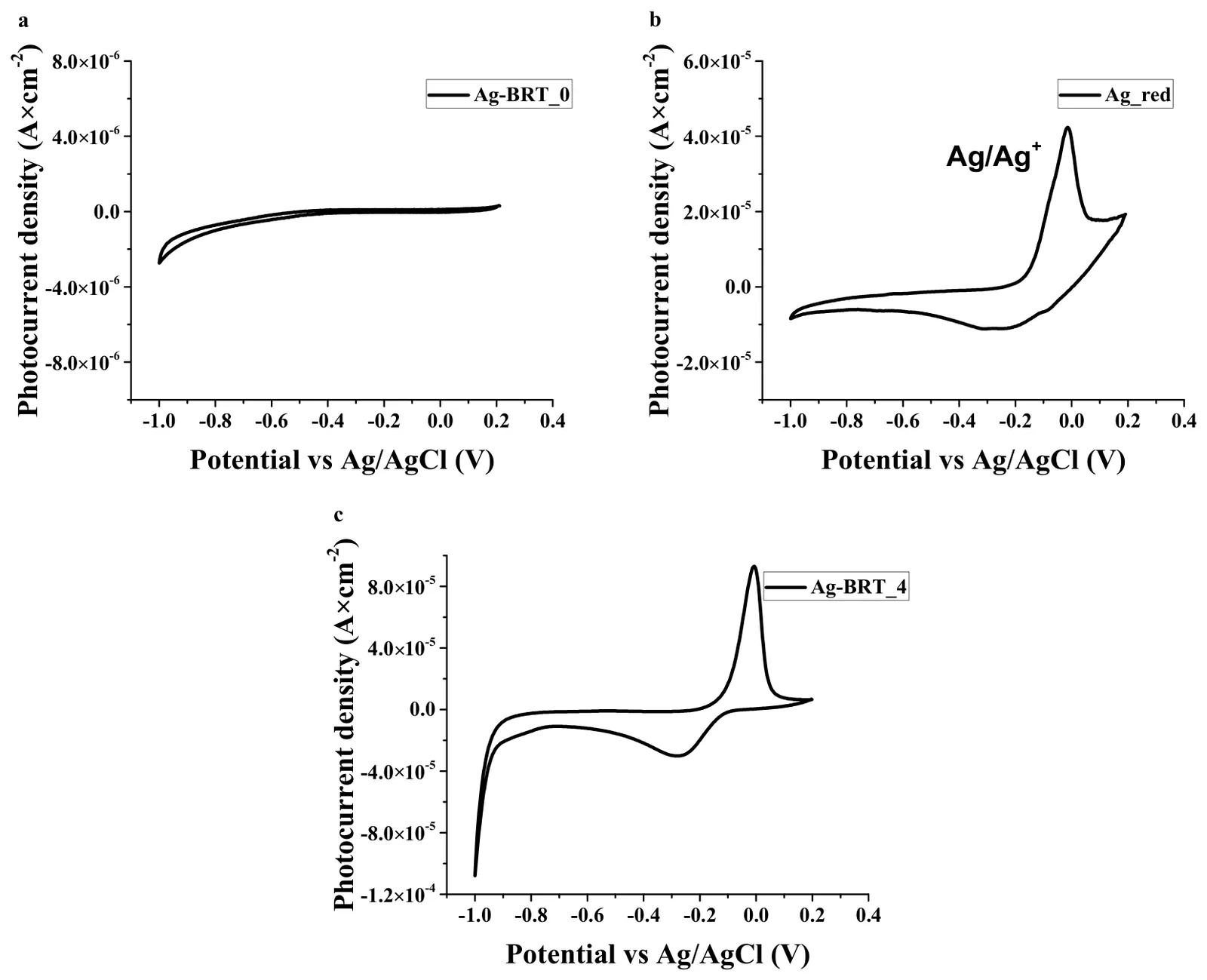
Cyclic voltammograms of Ag-BRT_0 (**a**), Ag-red (**b**), and Ag-BRT_4 (**c**) samples.

The photocurrent density results are shown in Figure 7. The photocurrent densities consistently decrease with increasing nominal Ag content. The decrease is due to silver deposition, as silver deposition reduces the free BaTiO_3_ surface area, and the shielding effect of the deposited silver. In BaTiO_3_, photocurrent is only possible through the oxidation of sulfite ions, as BaTiO_3_ is an n-type semiconductor. In n-type semiconductors, photogenerated electrons can only move toward the electrode due to band bending, but holes can be utilized on the surface in contact with the solution, where they can oxidize suitable electron donor components [36]. Since the photocurrent density decreased in all cases when silver was also present on the surface, it can be concluded that only BaTiO_3_ is responsible for sulfite oxidation and, thus, for the measured photocurrent density. This is because the photocurrent decrease is substantial in samples containing silver, and this would not be the case if BaTiO_3_ were capable of taking electrons from the silver nanoparticles (thus oxidizing them), because then the measured photocurrent would be greater than that for BaTiO_3_. The figure shows an increase in dark current as well as photocurrent for the Ag-BRT_1, Ag-BRT_2, and Ag-BRT_3 samples. This current is no longer photocurrent, but current resulting from the oxidation of silver, and it is particularly pronounced in these samples due to them having the highest silver content (based on the XRF results). To verify this, cyclic voltammetry measurements (in the dark) were performed on reduced silver (Ag-red), BaTiO_3_, and the Ag-BRT_4 sample. The results are summarized in Figure 8. No positive current could be measured on BaTiO_3_, meaning that no oxidation occurred in the dark, while oxidation could clearly be measured in the sample containing silver, which can only be due to its presence.

### 2.2. Photocatalytic Activity

**Figure 9:**
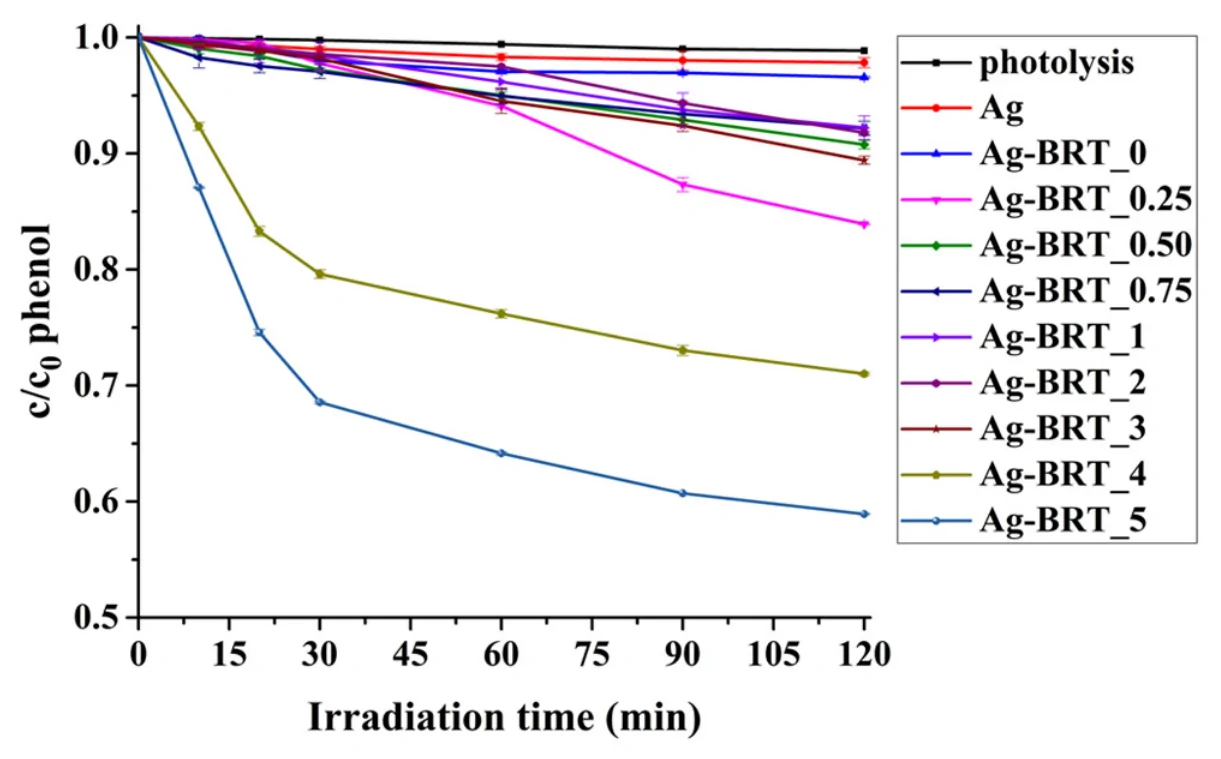
Photocatalytic oxidation of phenol using the investigated samples under UV light irradiation (c_catalyst_ = 1 g·L^−1^; c_phenol_ = 10 mg·L^−1^).

Each photocatalytic experiment was performed three times, and the error bars together with the degradation curves are shown in Figure 9. The photocatalyst suspension was stirred in the dark for 30 min to reach adsorption–desorption equilibrium. We found that phenol concentration changes are negligible since phenol adsorbs poorly. Photolysis cannot be observed either, meaning that the decreased concentrations result from photocatalytic processes. As shown in Figure 9, the commercial reference Ag-BRT_0 sample, which does not contain Ag, possesses the lowest photocatalytic activity, as expected. Increasing the nominal Ag amount also increases the photocatalytic activity, presumably due to the enhanced charge separation attributable to the BaTiO_3_ support, although the trend is inconsistent. Somewhat surprisingly, Ag-BRT_4 and Ag-BRT_5 are significantly better than the other samples. To determine the first-order reaction rate constants (k), the natural logarithm of the concentration was plotted as a function of time, in which case multiplying the slope by −1 gives k; the values are shown in Table S1. The values for Ag-BRT_4 and Ag-BRT_5 are one order of magnitude higher than those for the other silver-containing samples and reference BaTiO_3_. This could be because, for Ag-BRT_0.25–3 (i.e., Ag-BRT_0.25, Ag-BRT_0.5, Ag-BRT_0.75, Ag-BRT_1, Ag-BRT_2, and Ag-BRT_3), Ag nanoparticles presumably cover the surface tightly, decreasing the number of active sites available for photocatalytic reactions. The opposite is true for Ag-BRT_4 and Ag-BRT_5 due to the larger Ag particles. In these two samples, the larger silver particles do not cover the active sites of BaTiO_3_ significantly due to the relatively small contact area. Unlike during the photocurrent measurements, there is no potential applied to the system during the photocatalytic tests. In other words, phenol degradation is not carried out in an electrochemical cell but in a glass reactor containing the phenol solution. Thus, the silver is not oxidized during the measurement and therefore can exert its electron-trapping effect. Moreover, as previously noted, the deposited nanoparticles are significantly larger than those in the preceding members of the series, and larger particles are capable of trapping more electrons. For modified photocatalysts such as the one presented in this manuscript, charge separation is generally driven by some form of heterojunction, which is essential for efficient photocatalysis. In this case, a Type-II heterojunction is formed between BaTiO_3_ and Ag_2_O [37,38], which does not result in high photocatalytic efficiency under UV illumination. This is because UV irradiation primarily excites BaTiO_3_, and since the conduction band of BaTiO_3_ lies at a lower energy level than that of Ag_2_O, the photogenerated electrons are not transferred to the latter. The other heterojunction is a Schottky barrier formed between BaTiO_3_ and Ag, which operates such that the photogenerated electrons are easily transferred from the conduction band of BaTiO_3_ to the lower-lying Fermi-level of Ag, thereby achieving efficient charge separation. For Ag-BRT_4 and Ag-BRT_5, a higher amount of Ag is present on the surface (confirmed by TEM measurements), meaning that the effect of the Schottky barrier is more pronounced here. Due to the synthesis conditions, the actual Ag content decreases with an increase in the nominal Ag content because larger particles are formed, which sterically hinder each other’s deposition onto the surface, while larger particles yield more metallic silver, thus promoting Schottky barrier formation. Consequently, the highest photocatalytic activity is observed in Ag-BRT_5.

**Figure 10:**
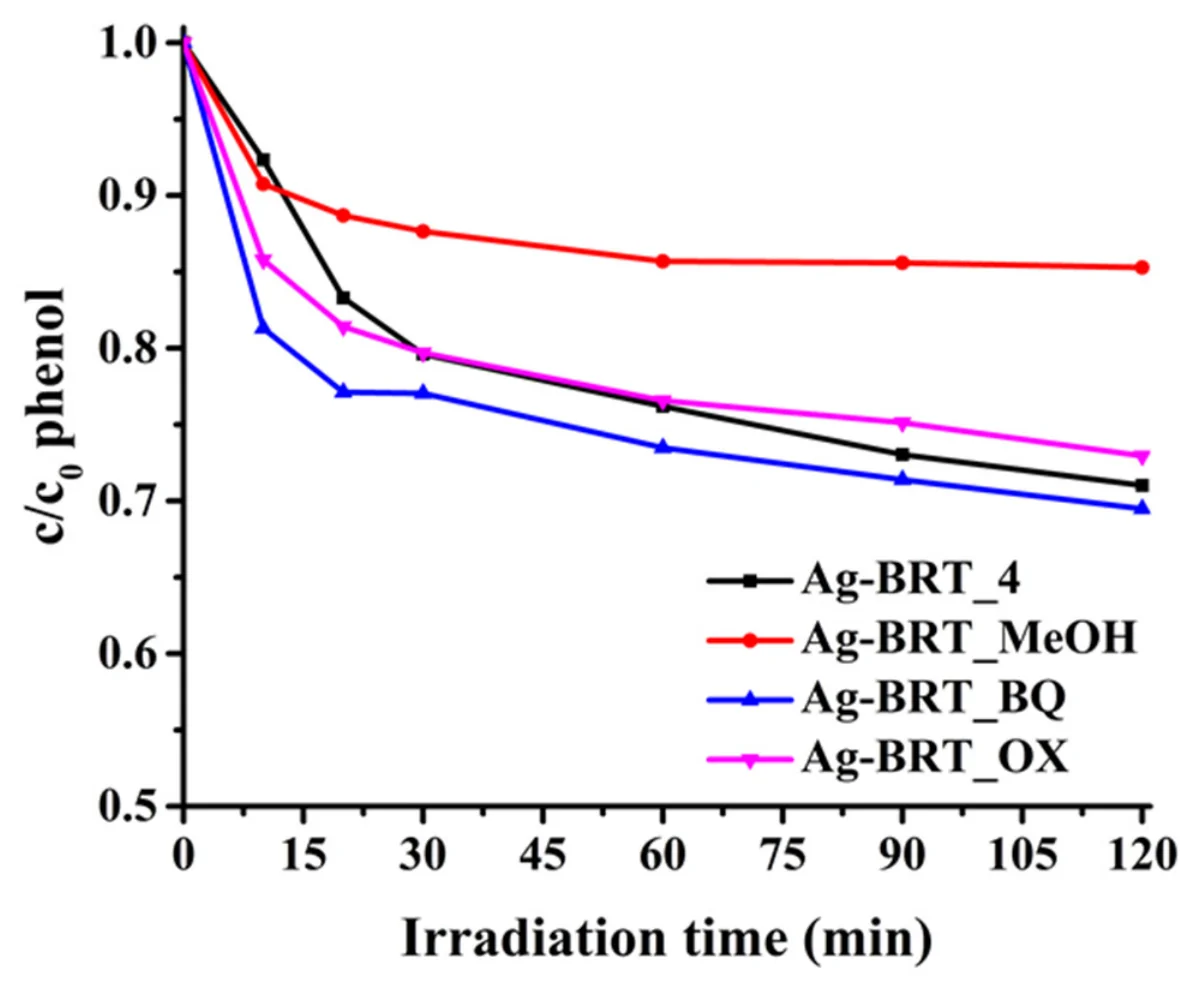
Photocatalytic degradation curves obtained in the presence of various radical scavengers.

For some additional measurements, the photocatalytic mechanism (Figure 10) and reusability (Figure S5) were also investigated. Methanol was used as a hydroxyl radical scavenger [39,40], benzoquinone was used as a superoxide radical scavenger [39], and oxalic acid was used as a hole scavenger [41]. Benzoquinone and oxalic acid did not significantly affect the photocatalytic activity of the tested photocatalyst (Ag-BRT_4), but methanol significantly reduced it (~15% difference in conversion). It can therefore be stated that phenol conversion mainly occurs via hydroxyl radicals under the conditions studied. Regarding reusability, photocatalytic activities decreased in the second and third cycles, as expected, which could be associated with the loss of catalysts and adsorption sites between the cycles. The first-order reaction rates are shown in Figure S6 and Table S1.

Based on the literature, a hypothetical decomposition pathway for phenol can be described as follows: photogenerated hydroxyl radicals attack the aromatic ring to yield hydroxylated intermediates (such as catechol, resorcinol, or hydroquinone), which can undergo further oxidation to trihydroxybenzenes and short-chain aliphatic carboxylic acids [42]. Ultimately, prolonged photocatalytic oxidation leads to complete mineralization into CO_2_ and H_2_O. It should be noted that, while our radical scavenger tests confirm hydroxyl radicals as the primary active species, the individual intermediate species and full mineralization were not directly quantified in this study.

## 3. Materials and Methods

### 3.1. Materials

Commercial BaTiO_3_ (ThermoFisher Scientific, 99.7%, Waltham, MA, USA) was used as a catalyst support. AgNO_3_ (Molar Chemicals, >99.9%, Halásztelek, Hungary), NaOH (Molar Chemicals, >97%, Halásztelek, Hungary), and NaBH_4_ (Alfa Aesar, 98%, Haverhill, MA, USA) were used to deposit silver nanoparticles on the catalyst surfaces. Ethanol (Molar Chemicals, 96%, Halásztelek, Hungary) and Milli-Q water were used for the purification of our Ag-modified BaTiO_3_ samples. Phenol (Spektrum 3D, analytical grade, Debrecen, Hungary) was used to evaluate photocatalytic activity.

### 3.2. Ag Deposition

An aqueous suspension of BaTiO_3_ (c = 50 g·L^−1^) was prepared first; then, a calculated amount of AgNO_3_ (nominal wt% = 0, 0.25, 0.5, 0.75, 1, 2, 3, 4, and 5) was added to the suspension. This was followed by the stochiometric dropwise addition of NaOH (c_stock_ = 8 g·L^−1^). As a result, an unstable AgOH precipitate was formed, which was converted into Ag_2_O. This conversion was proven by the color of the solution turning brown [43]. Last, a 10% excess of NaBH_4_ (c_stock_ = 4 g·L^−1^) was used to reduce all Ag_2_O to elementary Ag, whose appearance was confirmed by the pink color of the suspension. The products were washed three times with ethanol and three times with Milli-Q water via centrifugation. After drying and grinding in an agate mortar, the samples were stored in a dark and cool environment. The samples investigated in this study are referred to as “Ag-BRT_X”, where “BRT” stands for “barium titanate” and “X” stands for the nominal Ag amount in wt%.

### 3.3. Characterization

Crystalline compositions were investigated by X-ray diffraction (XRD; Rigaku Miniflex II, Rigaku, Neu-Isenburg, Germany) measurements under the following conditions: λ_Cu Kα_ = 0.15406 nm, 30 mA, 40 kV, 20–80 2θ° region, and 1 θ°·min^−1^ scan speed. The average primary crystal sizes were calculated using the Scherrer equation. The silver content on the surface of the catalysts was measured by X-ray fluorescence (XRF; Olympos VMR, Waltham, USA), using 10 and 40 keV excitation beams. Optical properties were evaluated by diffuse reflectance spectroscopy (DRS; Shimadzu UV-3600 Plus, Shimadzu Corporation, Kyoto, Japan), recording the absorbance spectra between 200 and 850 nm, and by photoluminescence (PL; Horiba Jobin Yvon Fluoromax-4, Horiba, Kyoto, Japan) at 375 nm excitation wavelength, using a high-pass filter cutoff (375 nm) and a band-pass filter (375 nm) before the monochromator of the detector and after the monochromator of the light source, respectively. The time-resolved fluorescence lifetime of the samples was investigated using a FluoroMax spectrofluorometer equipped with a DeltaHub TCSPC controller (Horiba Scientific, Piscataway, NJ, USA) and a laser light source (λ_max_ = 375 nm).

The morphology of the samples and the relative size of the deposited silver particles were studied by transmission electron microscopy (TEM; FEI TECNAI G2 20 X-Twin, FEI Company, Eindhoven, The Netherlands) measurements. To calculate the specific surface area, nitrogen adsorption measurements were carried out (with a Nova 3000e surface area analyzer). Electrochemical properties were examined by linear sweep voltammetry (LSV) measurements using a Metrohm Autolab PGSTAT302N potentiostat (Metrohm AG, Herisau, Switzerland) equipped with a three-electrode system in an aqueous medium. The voltammograms were recorded between −0.9 and 0.3 V potentials at a 5 mV·s sweeping rate. A 200 mW·cm^−2^ intensity was set for the light source. The working, counter, and reference electrodes consisted of fluorine-doped tin oxide (FTO)/photocatalysts, a platinum wire, and a Ag/AgCl wire submerged in a 3 mol·L^−1^ NaCl solution, respectively. The working electrodes were prepared by cutting 1 × 2.5 cm FTO plates and covering them with 10 mg·cm^−2^ Ag-BRT_X samples on a 1 cm^2^ surface via spray coating. All currents were normalized to the geometric surface area of the electrodes. A 0.5 mol·L^−1^ Na_2_SO_3_ solution was applied to ensure conduction and act as a hole scavenger. Figure S7 shows the emission spectrum of the light source used.

X-ray photoelectron spectroscopy (XPS) measurements were carried out to investigate the surface chemical environment with a SPECS PHOIBOS 150 MCD instrument, (Berlin, Germany) consisting of a monochromatic Al-Kα source (1486.6 eV), a hemispherical analyzer, and a charge neutralization device. The following parameters were used: 200 W X-ray source, 10^−9^–10^−10^ mbar chamber pressure, calibration of the binding energy scale to the C 1 s peak at 284.6 eV, and 60 and 20 eV pass energies for recording survey and high-resolution spectra, respectively. Data evaluation was performed in CasaXPS (version 2.3.1.), using a Shirley background and a 70% Gaussian and 30% Lorentzian product form for curve fitting.

### 3.4. Photocatalytic Experiments

The photocatalytic activity of the Ag-deposited BaTiO_3_ samples was evaluated by the decomposition of phenol under UV-A (λ_max_ = 364 nm; Figure S8) irradiation. The photocatalyst suspensions (c_catalyst_ = 1 g·L^−1^) containing phenol (c_phenol_ = 10 mg·L^−1^) were put into an open-top glass reactor, which was covered with a quartz plate to prevent evaporation. The suspensions were continuously stirred at 400 rpm during the 2-h-long measurements. The UV lamp was placed 5 cm above the suspensions. The suspensions were stirred in the dark for 30 min to reach adsorption–desorption equilibrium. Samples were taken every 10 min during the first 30 min and then every 30 min for the remainder of the experiment. Then, the samples were centrifuged at 14.500 rpm for 5 min and filtered with a 0.1 μm Millex-VV PVDF filter. Changes in the phenol concentrations were measured by high-performance liquid chromatography (HPLC; Agilent 1290 Infinity II, Agilent Technologies, Waldbronn, Germany). The chromatograph was equipped with a UV detector (λ_detection_ = 210 nm) and a C18 column (InfinityLab Poroshell 120 EC, single-bond, Agilent, Santa Clara, CA, USA). A 30/70 (*v*/*v*) acetonitrile/water mixture was applied as the eluent, using a flow rate of 0.5 mL·min^−1^. Each photocatalytic experiment was performed three times. Reusability of the samples was investigated by repeating photodegradation tests in three consecutive cycles. The photocatalytic mechanism was also investigated by carrying out the experiments in the presence of radical scavengers (methanol for hydroxyl radicals, benzoquinone for superoxide radicals, and oxalic acid for photogenerated holes). The concentration of the radical scavenger was in 50-fold excess relative to that of the pollutant (500 mg L^−1^).

## 4. Conclusions

This study presents a comprehensive investigation into the role of silver co-catalyst particle size in tailoring the functional properties of Ag-BaTiO_3_ composites. Based on structural analysis and photoelectrochemical and photocatalytic performance measurements, we reveal that exceeding a critical size threshold alters not only the optical properties, such as the loss of plasmonic behavior above 4 wt% nominal Ag (due to its size), but also fundamentally shifts the charge transfer dynamics. Higher precursor and reducing agent concentrations result in larger silver particles, which, through steric hindrance, can be used to optimize the trade-off between surface coverage and active site exposure. Acting as exceptional electron sinks, they drastically extend carrier lifetimes (reaching up to hundreds of microseconds) and suppress electron–hole recombination, leading to a tenfold enhancement in photocatalytic activity. The results presented in this work confirm that silver deposition on BaTiO_3_ is an efficient approach to increase its photoactivity.

## Figures and Tables

**Table 1:** Nominal and measured Ag content (wt%) and primary crystallite size of the Ag-BRT samples.

Sample Name	Nominal Ag Content (wt%)	Measured Ag Content (wt%)	Specific Surface Area (m^2^ g^−1^)	Primary Crystallite Size (nm)
Ag-BRT_0	0	0	4.61	27.05
Ag-BRT_0.25	0.25	0.22	3.56	27.26
Ag-BRT_0.5	0.5	0.46	3.69	28.55
Ag-BRT_0.75	0.75	0.69	3.94	27.22
Ag-BRT_1	1	1.13	3.74	28.19
Ag-BRT_2	2	1.9	4.16	27.99
Ag-BRT_3	3	2.58	3.26	28.53
Ag-BRT_4	4	1.45	2.33	28.67
Ag-BRT_5	5	1.22	2.45	28.22

## Data Availability

The original contributions presented in this study are included in the article/Supplementary Materials. Further inquiries can be directed to the corresponding author.

## Supplementary Materials

The following supporting information can be downloaded at: https://www.mdpi.com/article/10.3390/nano16201322/s1, Figure S1: Calculation of band gaps via Tauc plots; Figure S2: Goodness of fit of time-resolved PL measurements; Figure S3: TEM micrographs of the Ag-BRT sample series; Figure S4: BET plots of the investigated photocatalysts; Figure S5: Reusability of the photocatalysts; Figure S6: Determination of the first-order reaction rate constants (k); Figure S7: Emission spectrum of the light source used during the photocurrent measurements; Figure S8: Emission spectrum of the light source used during photocatalytic oxidation experiments; Table S1: First-order reaction rates of the photocatalysts calculated via reusability experiments.
