# Supplementary material for: Effect of Silver Deposition on the Material Characteristics and Photocatalytic Activity of Barium Titanates
Source: Nanomaterials (Basel). 2026 Oct 20;16(20):1322. doi: 10.3390/nano16201322 (PMC13708379; doi:10.3390/nano16201322)
Supplement: Supplementary file 1 [file nanomaterials-16-01322-s001.zip › nanomaterials-4586300-supplementary.pdf]

Effect of Silver Deposition on the Material Characteristics and Photocatalytic Activity of Barium Titanates

Áron Ágoston [1], Eszter Kanász [2], Szabolcs Kocsis Szürke [3], Mahsa Abedi [2], Karolina Solymos [4], Csaba Bús [5], Milica Todea [6,7], Ágota Deák [1], Tamás Gyulavári [2,*], Ákos Kukovecz [2], Zoltán Kónya [2] and Zsolt Pap [2,7,8]

[1] Department of Physical Chemistry and Materials Sciences, University of Szeged, Aradi v. Sqr. 1,
6720 Szeged, Hungary; agostona@chem.u-szeged.hu (Á.Á.); agotadeak@chem.u-szeged.hu (Á.D.)

[2] Department of Applied and Environmental Chemistry, University of Szeged, Rerrich Béla Sqr. 1,
6720 Szeged, Hungary; kanasz.eszter@gmail.com (E.K.); mahsa@chem.u-szeged.hu (M.A.);
kakos@chem.u-szeged.hu (Á.K.); konya@chem.u-szeged.hu (Z.K.); pzsolt@chem.u-szeged.hu (Z.P.)

[3] Vehicle Industry Research Center, Széchenyi István University, Egyetem tér 1. 303, Győr 9026, Hungary; kocsis.szurke.szabolcs@sze.hu

[4] Department of Physical and Environmental Geography, University of Szeged, Egyetem Str. 2-6,
6722 Szeged, Hungary; solymoskarolina@geo.u-szeged.hu

[5] Department of Molecular and Analytical Chemistry, University of Szeged, Dóm Sqr. 7-8, 6720 Szeged, Hungary; bcsaba0312@gmail.com

[6] Faculty of Nursing and Health Sciences, Iuliu Hațieganu University of Medicine and Pharmacy, Victor Babeș 8, RO-400012 Cluj-Napoca, Romania; militodea@yahoo.com

[7] Nanostructured Materials and Bio-Nano-Interfaces Center, Interdisciplinary Research Institute on Bio-Nano-Sciences, Babes-Bolyai University, Treboniu Laurian 42, RO–400271 Cluj-Napoca, Romania

[8] Institute of Research-Development-Innovation in Applied Natural Sciences, Babes-Bolyai University, Fântânele Str. 30, RO-400294 Cluj-Napoca, Romania

* Correspondence: gyulavarit@chem.u-szeged.hu

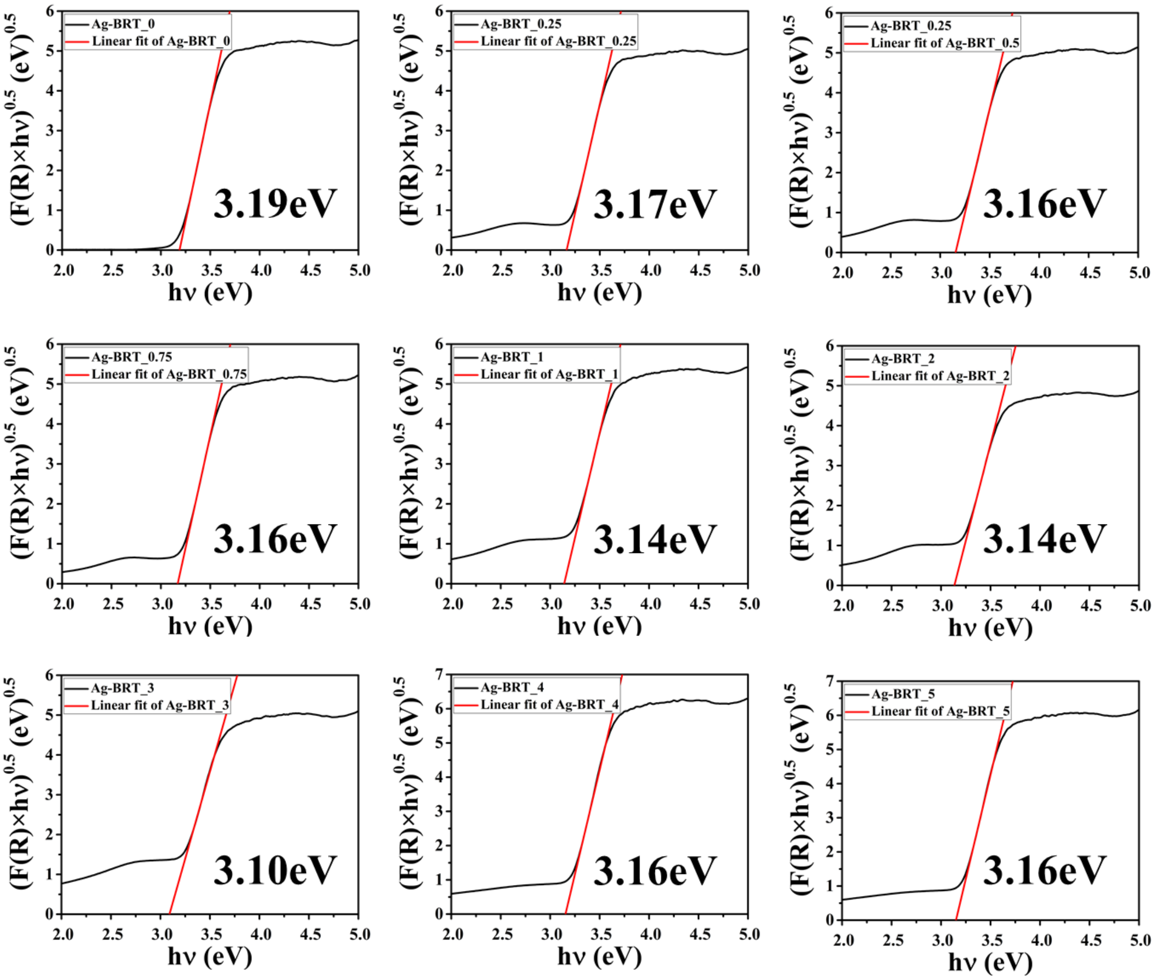

Figure S1. Calculation of band gaps via Tauc plots.

The fitting model follows the general formula of the multiexponential function given below:

$$y = a + \sum_{i=1}^{n} b_i \cdot e^{\frac{-t}{\tau_i}}$$

a: constant

b_i: i-th pre-exponential factor

t: time

τ_i: i-th fluorescence lifetime

The goodness of the fits can be estimated using the χ^2 value. For two components, the formula is as follows:

$$y = a + b_1 \cdot e^{\frac{-t}{\tau_1}} + b_2 \cdot e^{\frac{-t}{\tau_2}}$$

For Ag-BRT_0, Ag-BRT_0.5, and Ag-BRT_4, the fitting was performed using two components, meaning there are two pre-exponential factors, whereas for Ag-BRT_5, three components were used, resulting in three pre-exponential factors. The following table summarizes the chi2 values for the four measurements, the lifetimes of the components constituting the average lifetime, and their contributions to the average lifetime.

Parameter	Ag-BRT_0	Ag-BRT_0.5	Ag-BRT_4	Ag-BRT_5
Average lifetime (ns)	0.92	3.60	18.21	42.8
τ_1	5.11	566.96	2.57	2.83
τ_1 relative contribution (%)	4.31	47.94	13.87	6.31
τ_2	0.89	1.88	944.4	1525.0
τ_2 relative contribution (%)	95.69	52.06	86.13	76.75
τ_3	-	-	-	273.3
τ_3 relative contribution (%)	-	-	-	16.94
χ^2	0.795	0.965	1.302	1.183

The following figure shows the goodness of the fit (IRF), displaying the measured data set subtracted from the fitted curve.

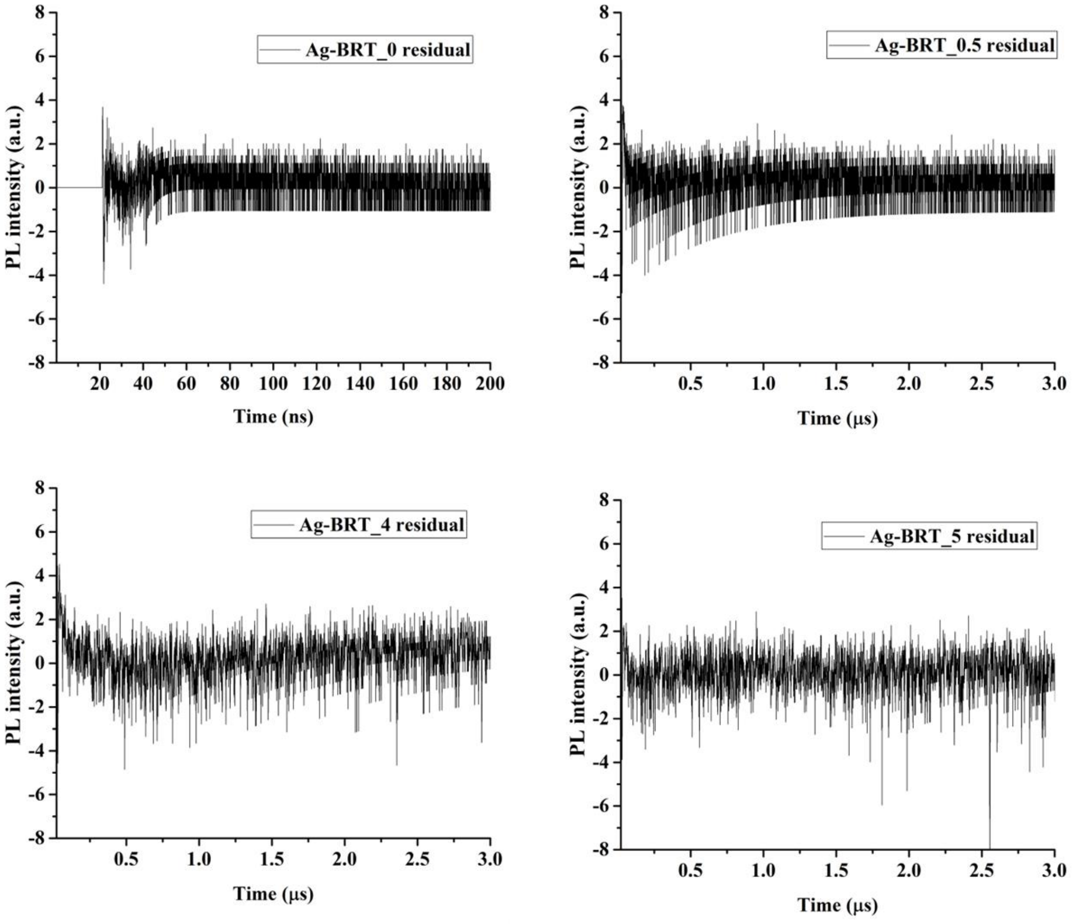

Figure S2 Goodness of fit of time-resolved PL measurements.

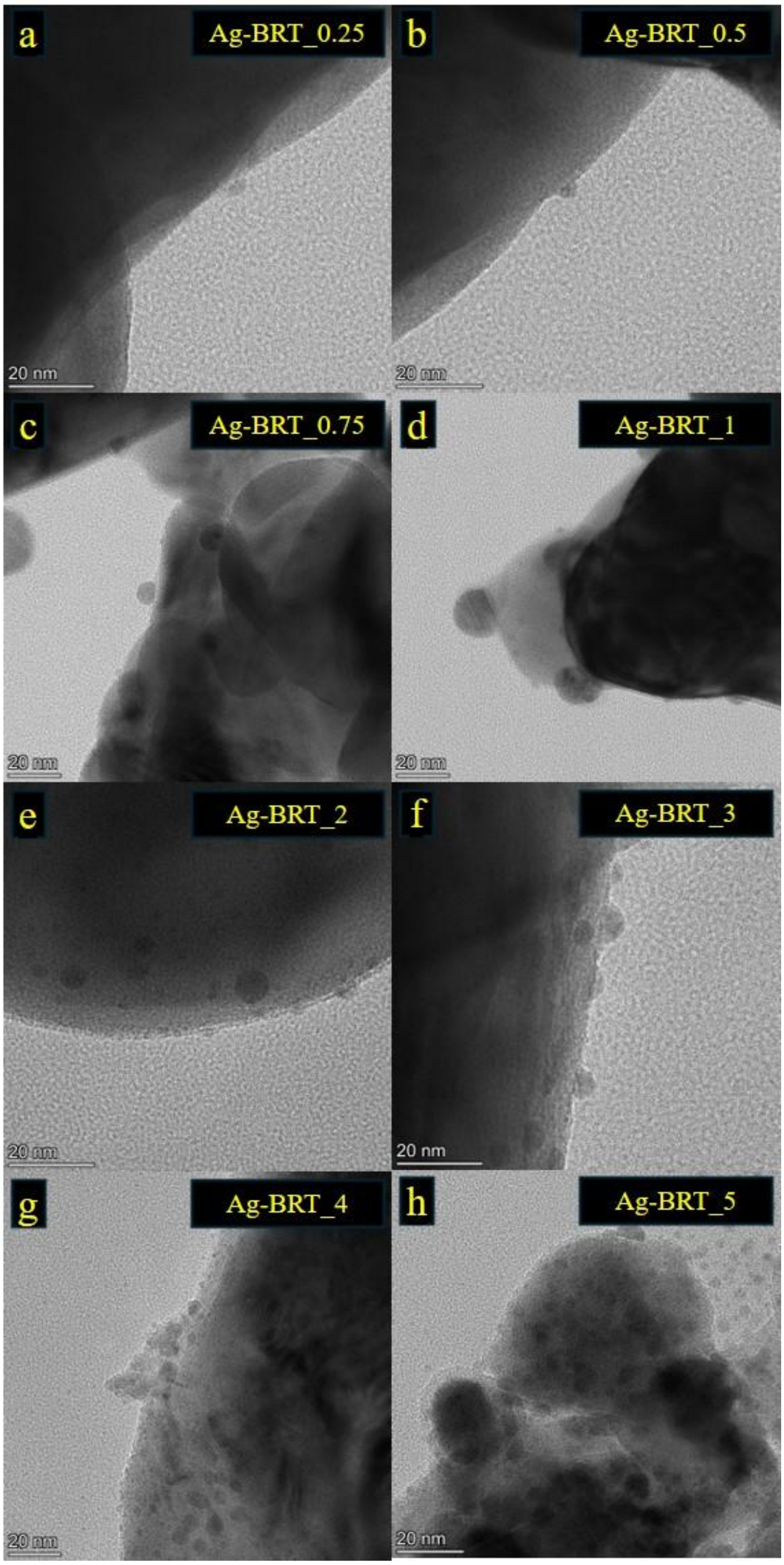

Figure S3 TEM micrographs of the Ag-BRT sample series.

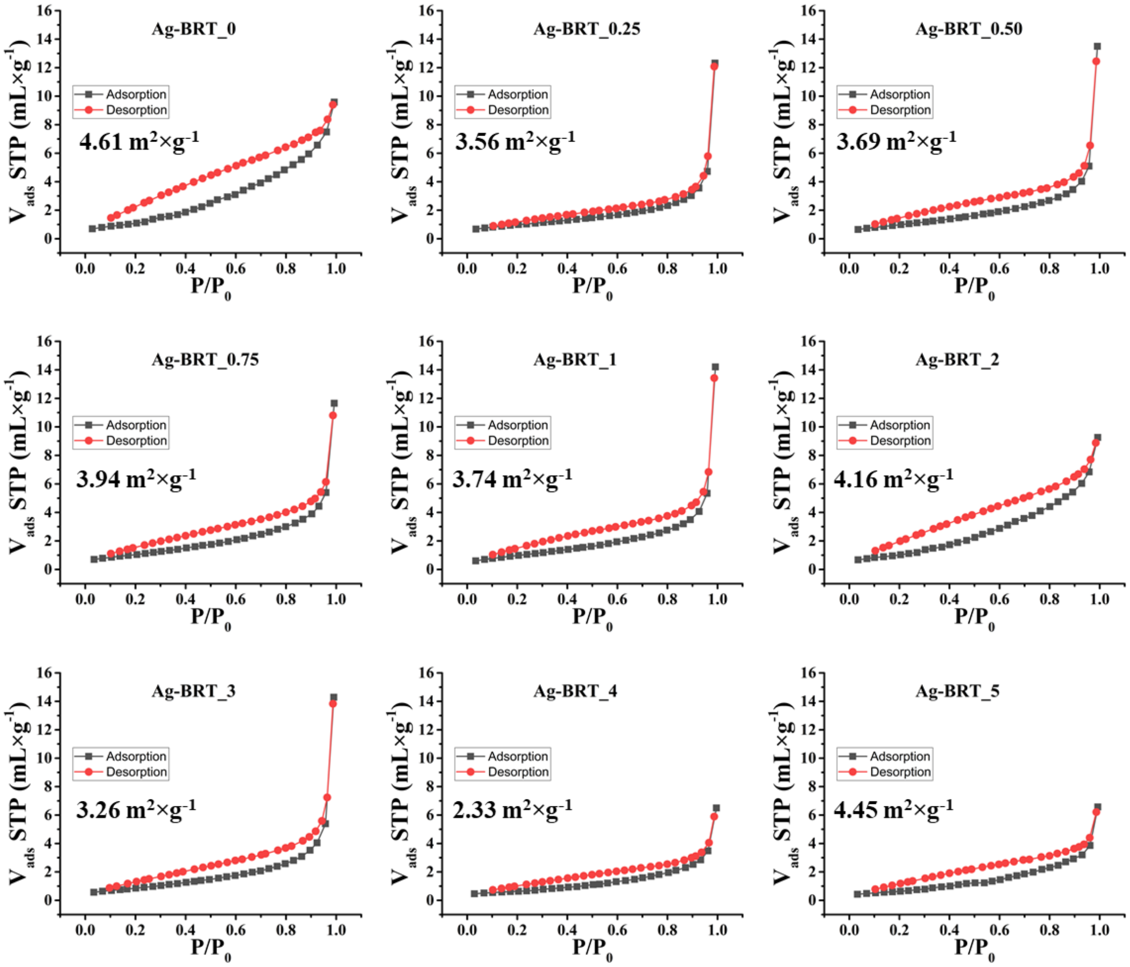

Figure S4 BET plots of the investigated photocatalysts.

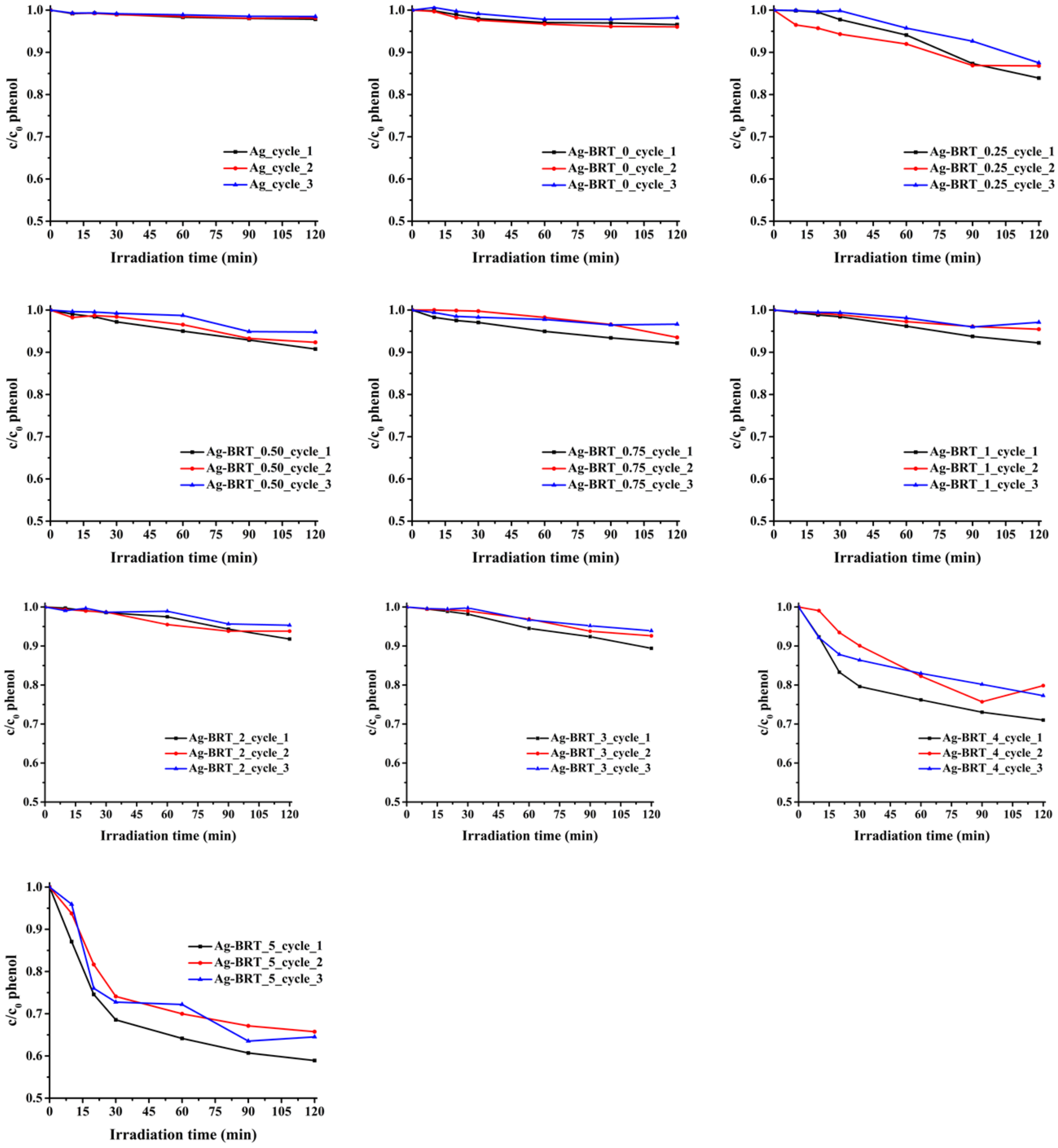

Figure S5. Reusability of the photocatalysts.

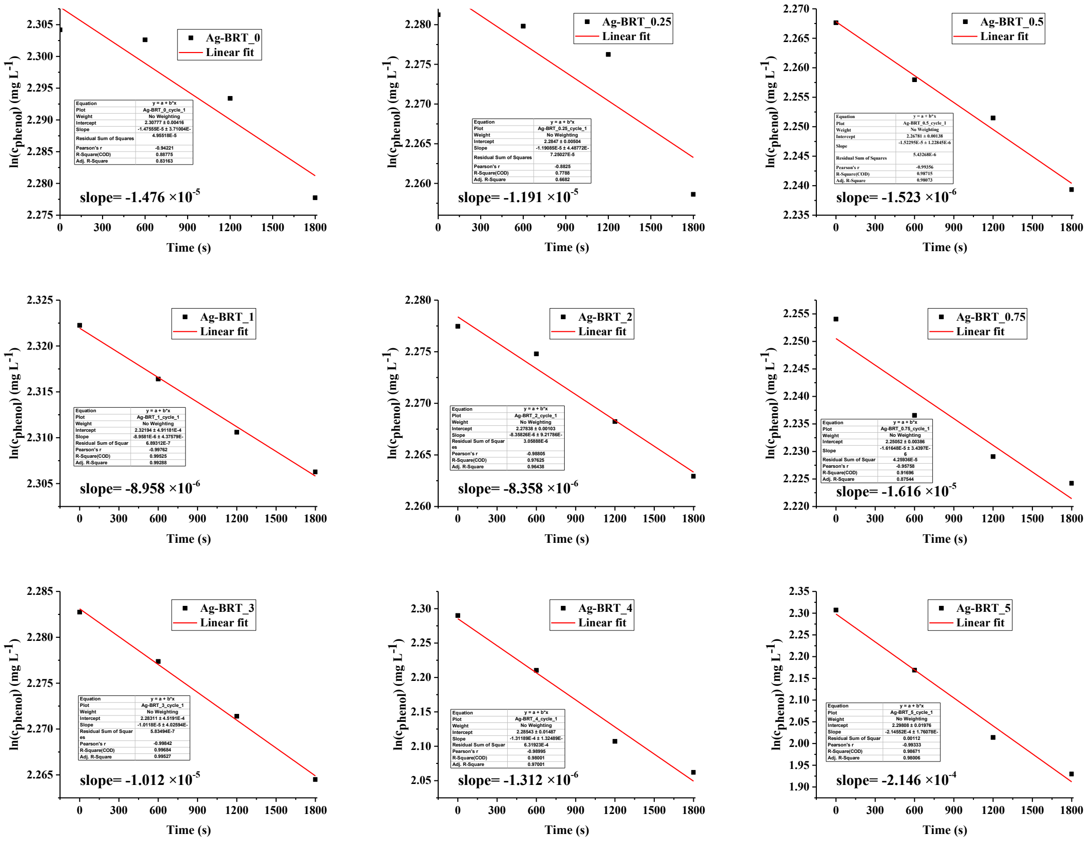

Figure S6. Determination of the first-order reaction rate constants (k).

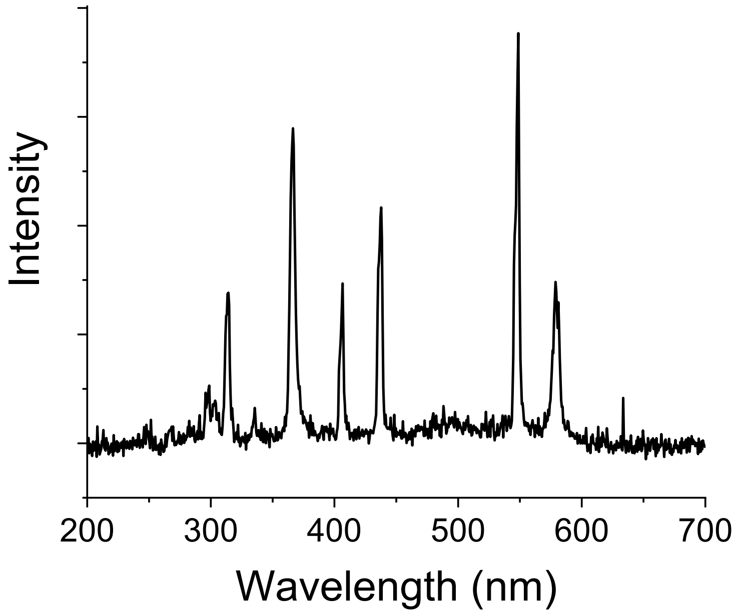

Figure S7. Emission spectrum of the light source used during the photocurrent measurements.

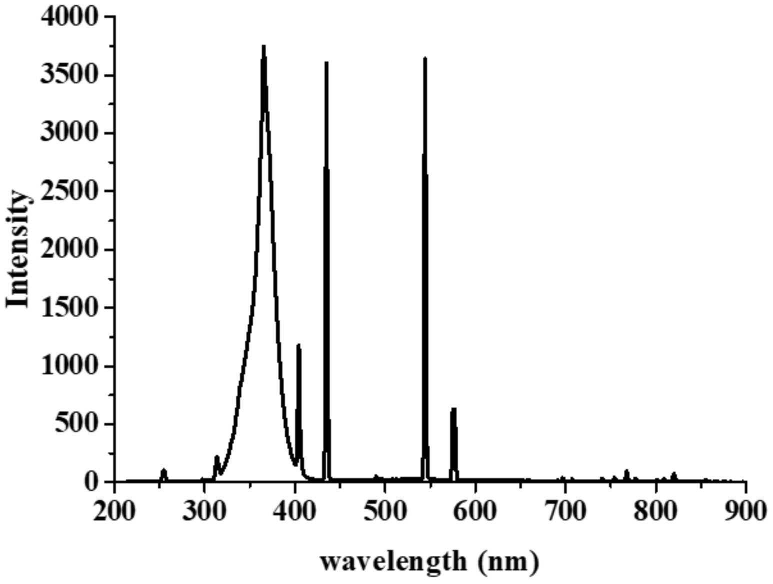

Figure S8. Emission spectrum of the light source used during photocatalytic oxidation experiments.

Table S1. First-order reaction rates of the photocatalysts calculated via reusability experiments.

Sample name	Cycle 1.	Cycle 2.	Cycle 3.
Ag-BRT_0	1.47×10^{-5}	1.33×10^{-5}	4.76×10^{-6}
Ag-BRT_0.25	1.26×10^{-5}	3.25×10^{-5}	8.03×10^{-6}
Ag-BRT_0.5	1.57×10^{-5}	8.94×10^{-6}	4.24×10^{-6}
Ag-BRT_0.75	1.66×10^{-5}	1.43×10^{-6}	9.53×10^{-6}
Ag-BRT_1	8.88×10^{-5}	6.16×10^{-6}	3.34×10^{-6}
Ag-BRT_2	8.07×10^{-6}	7.35×10^{-6}	7.47×10^{-6}
Ag-BRT_3	1.01×10^{-5}	5.66×10^{-6}	1.25×10^{-6}
Ag-BRT_4	1.27×10^{-4}	5.80×10^{-5}	8.13×10^{-5}
Ag-BRT_5	2.10×10^{-4}	1.67×10^{-4}	1.77×10^{-4}
